# Estimating the frequency of causal genetic variants in foetuses with congenital heart defects: a Chinese cohort study

**DOI:** 10.1186/s13023-021-02167-8

**Published:** 2022-01-04

**Authors:** Fengying Lu, Peng Xue, Bin Zhang, Jing Wang, Bin Yu, Jianbin Liu

**Affiliations:** 1 Department of Medical Genetics, Changzhou Maternity and Child Health Care Hospital, Nanjing Medical University, Changzhou, 213000 China; 2 Changzhou Children’s Hospital of Nantong Medical University, No. 468, Yanling East Road, Changzhou, 213003 Jiangsu Province China

**Keywords:** Congenital heart defects, Chromosomal abnormalities, Copy number variations, Chromosome microarray analysis, Whole exome sequencing

## Abstract

**Background:**

The belief that genetics plays a major role in the pathogenesis of congenital heart defects (CHD) has grown popular among clinicians. Although some studies have focused on the genetic testing of foetuses with CHD in China, the genotype–phenotype relationship has not yet been fully established, and hotspot copy number variations (CNVs) related to CHD in the Chinese population are still unclear. This cohort study aimed to assess the prevalence of chromosomal abnormalities in Chinese foetuses with different types of CHD.

**Results:**

In a cohort of 200 foetuses, chromosomal abnormalities were detected in 49 (24.5%) after a prenatal chromosome microarray analysis (CMA), including 23 foetuses (11.5%) with aneuploidies and 26 (13.0%) with clinically significant CNVs. The additional diagnostic yield following whole exome sequencing (WES) was 11.5% (6/52). The incidence of total chromosomal abnormality in the non-isolated CHD group (31.8%) was higher than that in the isolated CHD group (20.9%), mainly because the incidence of aneuploidy was significantly increased when CHD was combined with extracardiac structural abnormalities or soft markers. The chromosomal abnormality rate of the complex CHD group was higher than that of the simple CHD group; however, the difference was not statistically significant (31.8% vs. 23.6%, P = 0.398). The most common CNV detected in CHD foetuses was the 22q11.2 deletion, followed by deletions of 5p15.33p15.31, deletions of 15q13.2q13.3, deletions of 11q24.2q25, deletions of 17p13.3p13.2, and duplications of 17q12.

**Conclusions:**

CMA is the recommended initial examination for cases of CHD in prenatal settings, for both simple heart defects and isolated heart defects. For cases with negative CMA results, the follow-up application of WES will offer a considerable proportion of additional detection of clinical significance.

**Supplementary Information:**

The online version contains supplementary material available at 10.1186/s13023-021-02167-8.

## Background

Congenital heart defects (CHD) are the most common congenital defects, occurring in 5–8 in 1,000 live births [1, 2]. The occurrence of CHD is related not only to genetic factors but also to some maternal factors, such as maternal infection with rubella virus, radiation, drug use, and environmental pollution [3, 4]. Recently, an increasing number of clinicians believe that genetics plays a central role in CHD pathogenesis. Known genetic causes include chromosomal abnormalities, chromosome copy number variations (CNVs), and mutations of heart-related genes [5]. Chromosomal abnormalities were thought to be the most common causes of CHD [6, 7],

of which aneuploidies were the earliest identified and most common [8]. Trisomy 13, trisomy 18, trisomy 21, and Turner syndrome have also been confirmed to be associated with CHD [9]. Clinically significant CNVs have been detected in approximately 10%–15% of patients with CHD [10]. Notably, the deletions at 22q11.2 in DiGeorge syndrome were presented in approximately 2% of CHD patients and 13% of patients with specific cardiac malformations. However, CNVs and aneuploidy may only account for ~ 23% of CHD, overall. With the advances in sequencing technologies, genes associated with CHD have been discovered. It has been confirmed that more than 400 gene variants could cause human CHD. Genes such as, *NKX2-5*, *TBX5*, *GATA6*, *CHD7*, *NOTCH1*, etc. affect various aspects of cardiac development and function [11]. Likewise, a chromosome microarray analysis (CMA) can detect both chromosomal aberrations and CNVs at the genome-wide level. Currently, the CMA has been clinically recommended as the preferred cytogenetic diagnostic test for CHD [12]. The rapid development of next-generation sequencing technologies, particularly whole exome sequencing (WES), has uncovered mutations that cannot be defined by traditional genomic approaches, thereby facilitating the understanding of the genetics of complex diseases, such as CHD [13].

Recently, there have been some studies focused on the genetic testing of foetuses with CHD in China. Still, the genotype–phenotype relationship has not yet been fully established, and the hotspot CNVs related to CHD in the Chinese population remain unclear. Here, we present the CMA analysis of 200 CHD foetuses to evaluate the diagnostic effect of CMA for the prenatal diagnosis of CHD and investigate the possible genetic causes of prenatal CHD cases. Further, we aimed to identify new genes associated with CHD through WES and also explore the clinical value of WES in prenatal diagnosis. We also performed a systematic literature search to investigate hotspot pathogenic CNVs associated with CHD in the Chinese population. Our study further aimed to provide the basis for the standard method of gene detection in CHD foetuses.

## Results

### Aetiology of CHD

Among the 200 foetuses included in the CMA test, 134 presented with isolated CHD and 66 presented with non-isolated CHD, including structural anomalies (n = 28), soft markers (n = 22), and structural anomalies and soft markers (n = 16). Of the 200 foetuses, 178 presented with simple CHD and 22 with complex CHD. Further, according to the anatomical classification proposed by Botto et al. [14], participants were divided into eight main groups. Among them, the three most common heart abnormalities were septal defects (60/200, 30.0%), conotruncal defects (49/200, 24.5%), and left ventricular outflow tract defects (29/200, 14.5%).

**Fig. 1:**
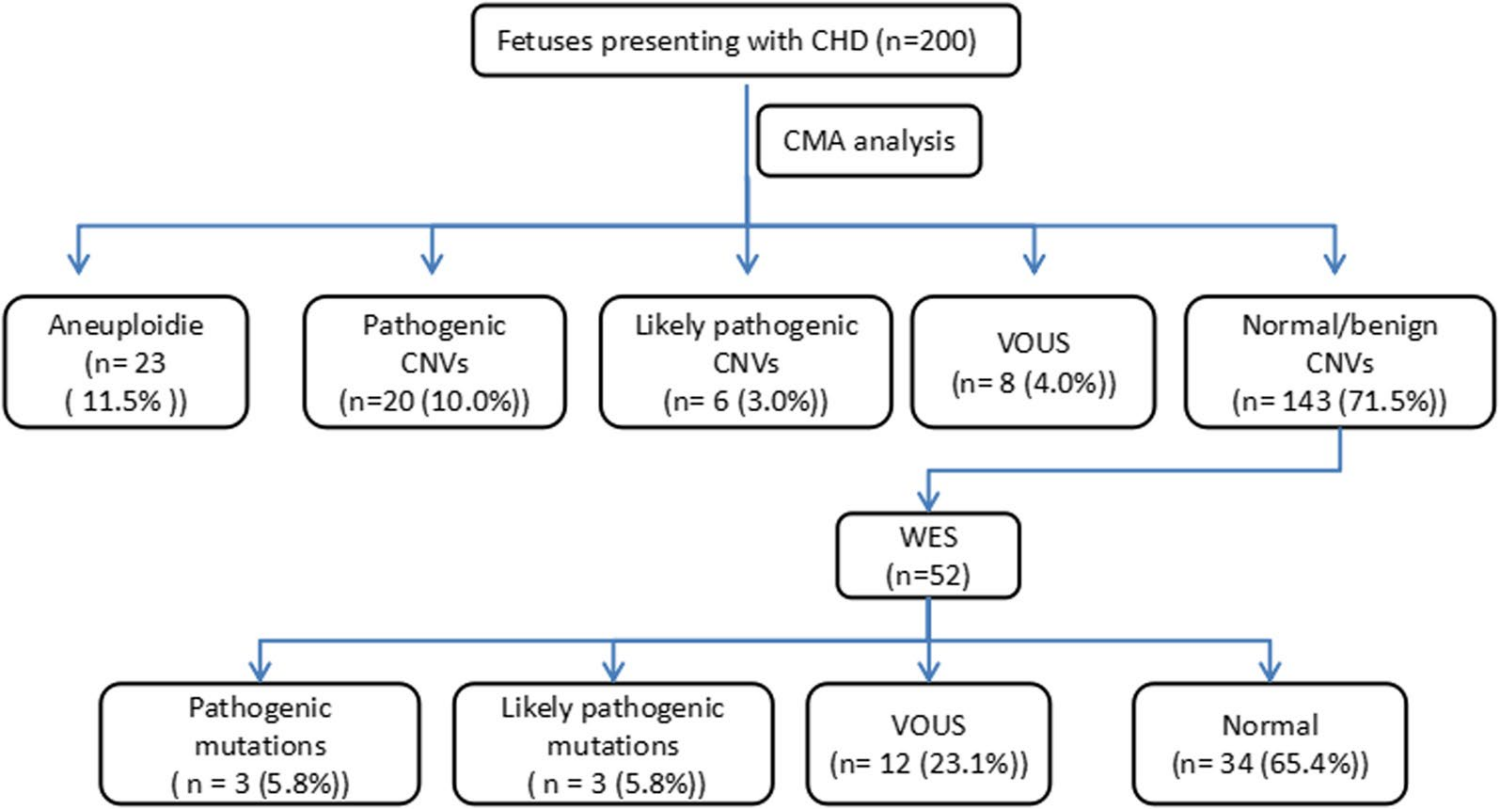
Flow chart of the study and results overview. CHD: congenital heart disease; CMA: Chromosomal microarray analysis; CNVs, copy number variations; VOUS, variants of uncertain significance

After prenatal CMA testing, chromosomal abnormalities were detected in 49 foetuses, the prevalence was 24.5% (49/200). Among them, 23 cases (11.5%) were of aneuploidies, including 8 cases of trisomy 21, 9 of trisomy 18, and 6 of trisomy 13. Additionally, clinically significant CNVs were detected in 26 (13%) cases, including 20 (10.0%) pathogenic (P) CNVs and 6 (3.0%) likely pathogenic (LP) CNVs. Further, CNVs which were variants of unknown significance (VOUS) were detected in 8 (4%) cases; the other 143 foetuses were reported to presented with negative CMA results. Finally, 52 cases were recalled and received WES testing after genetic counselling, of which 6 (11.5%) were found to have P or LP sequence variants. The process and brief results of the study are shown in Fig. 1.

### Subgroup analysis of different types of CHD

Next, we evaluated the relationship between chromosomal abnormalities and CHD types. Compared with the isolated CHD group, the chromosomal abnormality rate and aneuploidy rate of the non-isolated CHD group were higher (31.8% vs 20.9%). However, the clinically significant CNVs rate was lower (Table 1, 12.1% vs 13.4%). It is worth noting that the higher rate of chromosomal abnormality in the non-isolated CHD group was mainly because the incidence of aneuploidy was significantly increased when CHD was combined with extracardiac structural abnormalities or soft markers (19.7% vs 7.5%).

**Table 1:** Distribution of genetic variants in different group

CHD group	n	Chromosomal abnormalities		
		Aneuploidies	Clinical significant CNVs	Total
Isolated CHD	134	10 (7.5%)	18 (13.4%)	28 (20.9%)
Non-isolated CHD	66	13 (19.7%)*	8 (12.1%)	21 (31.8%)
CHD plus structural anomalies	28	3(10.7%)	5(17.9%)	8(28.6%)
CHD plus soft marker	22	5(22.7%)*	1(4.5%)	6(27.3%)
CHD plus structural anomalies & soft marker	16	5(31.3%)*	2(12.5%)	7(43.8%)
Simple CHD	178	20(11.2%)	22(12.4%)	42(23.6%)
Complex CHD	22	3(13.6%)	4(18.2%)	7(31.8%)

*Compared with isolated CHD, p < 0.05

CHD, congenital heart disease; CNV, copy number variation;

Further, the chromosomal abnormality rate of the complex CHD group was higher than that of the simple CHD group (31.8% vs. 23.6%), including aneuploidy rates (13.6% vs. 11.2%) and clinically significant CNV rates (18.2% vs. 12.4%). Also, the incidence of foetal chromosomal abnormality was highest in foetuses with atrioventricular septal defects (AVSD) (54.5%). The chromosomal abnormality rates of different subgroups of CHD are shown in Table 2.

**Table 2:** Types of congenital heart disease and frequencies for fetuses with chromosomal abnormalities

CHD classfication	No. tested	Aneuploidies	significant CNVs	Total	DR (%)
**Septal defects**	**60**	**7**	**5**	**12**	**20.0**
VSD	55	6	4	10	18.2
VSD + ASD	5	1	1	2	40.0
**Conotruncal defects**	**49**	**4**	**9**	**12**	**24.5**
Truncus arteriosus	4	0	0	0	0
IAA	8	0	2	2	25.0
d-TGA	8	0	1	1	12.5
DORV	11	1	2	2	18.2
TOF	18	3	4	7	38.9
**LVOTO**	**29**	**4**	**3**	**7**	**24.1**
HLHS	11	1		1	9.1
Coarctation of aorta	10	3	2	5	50.0
Aortic stenosis	8	0	1	1	12.5
**RVOTO**	**19**	**1**	**3**	**4**	**21.1**
Pulmonary stenosis	12	1	2	3	25.0
Pulmonary atresia	2	0	1	1	50.0
Tricuspid atresia	5	0	0	0	0
**AVSD**	**11**	**4**	**2**	**6**	**54.5**
**Complex CHD**	**22**	**3**	**4**	**7**	**31.8**
Multiple, complex heart anomaly	19	3	4	7	36.8
Single ventricle	2	0	0	0	0
L-TGA	1	0	0	0	0
**Heterotaxy**	**2**	**0**	**0**	**0**	**0**
**Other CHD**	**8**	**0**	**0**	**1**	**12.5**
**Total**	**200**	**23**	**26**	**49**	**24.5**

Bold indicates a major category

CHD, congenital heart disease; NCA, numerical chromosomal abnormality; DR, detection rate; CNV, copy number variation; pCNV, pathogenic copy number variation;VSD, ventricular septal defect; ASD, atrial septal defect; AVSD, atrioventricular septal defect; DORV, double outlet right ventricle; d-TGA, d-transposition of great arteries; IAA, interrupted aortic arch; LVOTO, left ventricular outflow tract obstruction; RVOTO, right ventricular outflow tract obstruction

Subsequently, we evaluated the association between the incidence of chromosomal abnormalities and extracardiac structural abnormalities. A total of 44 foetuses with extracardiac structural abnormalities were included, including 36 with single extracardiac structural abnormality and 8 with multiple extracardiac structural abnormalities. Overall, the incidence of chromosomal abnormality was 34.1% (15/44), aneuploidies were found in 18.2% (8/44), and clinically significant CNVs were found in 15.9% (7/44). Additionally, we found that there was no statistical difference between single extracardiac structural abnormality and multiple extracardiac structural anomalies (33.3% vs. 37.5%, p > 0.05). Moreover, among these cases of CHD with extracardiac structural abnormalities, those with central nervous system abnormalities presented with a high probability of chromosomal abnormalities. Table 3 summarizes the detection of chromosomal abnormalities in cases of CHD with different types of extracardiac structural abnormalities.

**Table 3:** Detection rates of chromosomal abnormalities in fetuses with CHD plus additional structural anomalies

CHD with additional structural anomalies	n	Chromosomal abnormalities		
		Total	Aneuploidy	pCNV
**CHD with single additional structural anomaly**	**36**	**12**	**6**	**6**
Central nervous system	3	2	1	1
Gastrointestinal system	2	1	1	0
Urinary tract system	4	1	0	1
Respiratory system	2	0	0	0
Skeletal system	10	4	3	1
Face	8	3	1	2
Cystic hygroma	6	1	0	1
Abdominal wall	1	0	0	0
**CHD with multiple additional structural anomalies**	**8**	**3**	**2**	**1**

Bold indicates a major category

Finally, among the 38 cases of CHD with soft markers, 34 presented with single soft markers and 4 with multiple soft markers. CHD combined with single umbilical artery and absent/shortened nasal bone were the most common. The incidence rate of chromosomal abnormality was 45.7% (13/38) in CHD foetuses with soft markers. The incidence of aneuploidy was (26.3%, 10/38) higher than that of clinically significant CNVs (7.9%, 3/38) in CHD foetuses with soft markers. Our data suggest that there was a high chance of detecting aneuploidies in CHD foetuses with soft markers, especially those with absent or shortened nasal bone (71.4%, 5/7). Moreover, combining multiple soft markers did not increase the chromosomal abnormalities (35.3% vs. 25%, p > 0.05). The chromosomal abnormalities of CHD with soft markers are shown in Table 4. In addition, no significant difference was observed in the chromosomal abnormality rates between CHD with extracardiac structural anomalies and CHD with soft markers groups (34.1% vs. 45.7%, *p* = 0.991). Notably, the chromosomal abnormality rates of the CHD combined with only soft markers group, the CHD combined with only structural anomalies group, and the CHD combined with both soft markers and structural anomalies group were 27.3%, 28.6%, and 43.8%, respectively. This suggests that the incidence of chromosomal abnormality was greatly increased in CHD foetuses presenting with both soft markers and additional structural anomalies (Table 1).

**Table 4:** Detection rates of chromosomal abnormalities in fetuses with CHD plus soft markers

CHD with nonstructuralanomalies	n	Chromosomal abnormalities		
		Total	Aneuploidy	pCNV
**CHD with single soft marker**	**34**	**12**	**9**	**3**
Single umbilical artery	11	3	1	2
Absent or shortened nasal bone	7	5	5	0
Mild ventriculomegaly	4	0	0	0
Short long bones	1	0	0	0
Echogenic bowel	1	1	0	1
Persistent right umbilical vein	3	0	0	0
Thickened nuchal fold	0	0	0	0
Enlarged cisterna magna	1	0	0	0
Increased nuchal translucency	0	0	0	0
Choroid plexus cysts	6	3	3	0
Pyelectasis	0	0	0	0
**CHD with multiple soft markers**	**4**	**1**	**1**	**0**

Bold indicates a major category

### WES analysis

After informed consent was obtained, 52 CHD foetuses with negative CMA tests were further analysed using WES, including 44 cases of isolated CHD and 8 cases of non-isolated CHD. As shown in Table 5, a total of 18 cases with 22 sequence variants which fulfilled the filtering criteria were detected. Three (5.8%) cases with pathogenic sequence variants and 3 (5.8%) cases with likely pathogenic sequence variants. The additional diagnostic yield of clinically significant sequence variants by WES testing for foetuses with CHD was 11.5% (6/52). Frequently encountered genes included *NOTCH1, GLI3, DNAH, SCN5A*.

**Table 5:** Detection of variants in fetuses with CHD using WES

Case	Ultrasound findings	Additional anomalies	Gene	Nucleotide change	Amino acid change	Zygosity	Clinical classify	Inherited mode	Disease
#133	RAA	–	*GLI3*	c.2308delG	p.A770fs	Het	LP	AD	Pallister-Hall syndrome
#79	AVSD	–	*SCN5A*	c.4357C > T	p.Q1453X	Het	LP	AD	Brugada syndrome 1
#25	VSD	Urinary tract system,Skeletal system	*NIPBL*	c.5220delA	p.T1740fs	Het	P	AD	Cornelia de Lange syndrome 1
#159	IAA, AVSD	–	*FOXF1*	c.853_854delAT	p.Ile285fs	Het	P	AD	Persistent fetal circulation syndrome
#170	VSD	–	*SCN5A*	c.362G > A	p.Arg121Gln	Het	P	AD	Brugada syndrome 1
#166	PA	–	*GATA6*	c.551G > A	p.Ser184Asn	Het	LP	AD	Atrial septal defect 9
#62	VSD,d-TGA,TA	–	*MYH7*	c.4076G > A	p.R1359H	Het	VUS	AD	–
#19	VSD,CA	–	*CITED2*	c.589A > G	p.S197G	Het	VUS	AD	–
#132	VSD	Mild ventriculomegaly	*NOTCH1*	c.7171C > T	p.Q2391X	Het	VUS	AD	–
#161	AS	–	*NOTCH1*	c.5339_5346dupAGAAGAAG	p.Glu1785fs	Het	VUS	AD	–
#168	VSD	–	*COL9A3*	c.622G > A	p.Gly208Ser	Het	VUS	AD	–
#171	TOF	–	*PRNP*	c.622C > T	p.Arg208Cys	Het	VUS	AD	–
#192	VSD	–	*HDAC*	c.1 + 1G > A		Het	VUS	AD	–
#194	Heterotaxy	Cystic hygroma	*DNAH9*	c.1997G > T	p.Trp666Leu	Het	VUS	AD	–
#200	Heterotaxy	–	*DNAH9*	c.14_34delAGGAGCGGGCCGCGC	p.Glu5_Ala11del	Het	VUS	AD	–
#98	TOF	–	*CCD22*	c.664C > T	p.R222W	Het	VUS	AD	–
#106	VSD, CA	–	*PRKD1*	c.2569G > C	p.E857Q	Het	VUS	AD	–
		Skeletal system,	*MYH6*	c.4036C > T	p.R1346W	Het	VUS	AD	–
		–	*COL5A1*	c.2768C > T	p.P923L	Het	VUS	AD	–
#51	HLHS	–	*GLI3*	c.2587C > T	p.R863C	Het	VUS	AD	–
#101	VSD	Thickened nuchal fold	*SOS2*	c.3812C > T	p.P1271L	Het	VUS	AD	–

Het, heterozygous; AD, autosomal dominant; AR, autosomal recessive; LP, likely pathogenic; P, pathogenic; VUS, variants of uncertain significance. VSD, ventricular septal defect; TGA, transposition of the great arteries; CA, coarctation of aorta; RAA, right aortic arch; AVSD, atrioventricular septal defect; TOF, tetralogy of fallot; HLHS, hypoplastic left heart syndrome; IAA, interruption of aortic arch; AS, aortic stenosis; PA, pulmomary stenosis; transposition of the great arteries; CA, coarctation of aorta; RAA, right aortic arch; AVSD, atrioventricular septal defect; TOF, tetralogy of fallot; HLHS, hypoplastic left heart syndrome; IAA, interruption of aortic arch; AS, aortic stenosis; PA, pulmomary stenosis

### Hotspot significant CNVs related to CHD in the Chinese population

In order to explore the characteristics of clinically significant CNVs associated with CHD in the Chinese population, we also conducted a systematic literature review. Five papers which met our criteria were selected for a detailed full-text review.

**Fig. 2:**
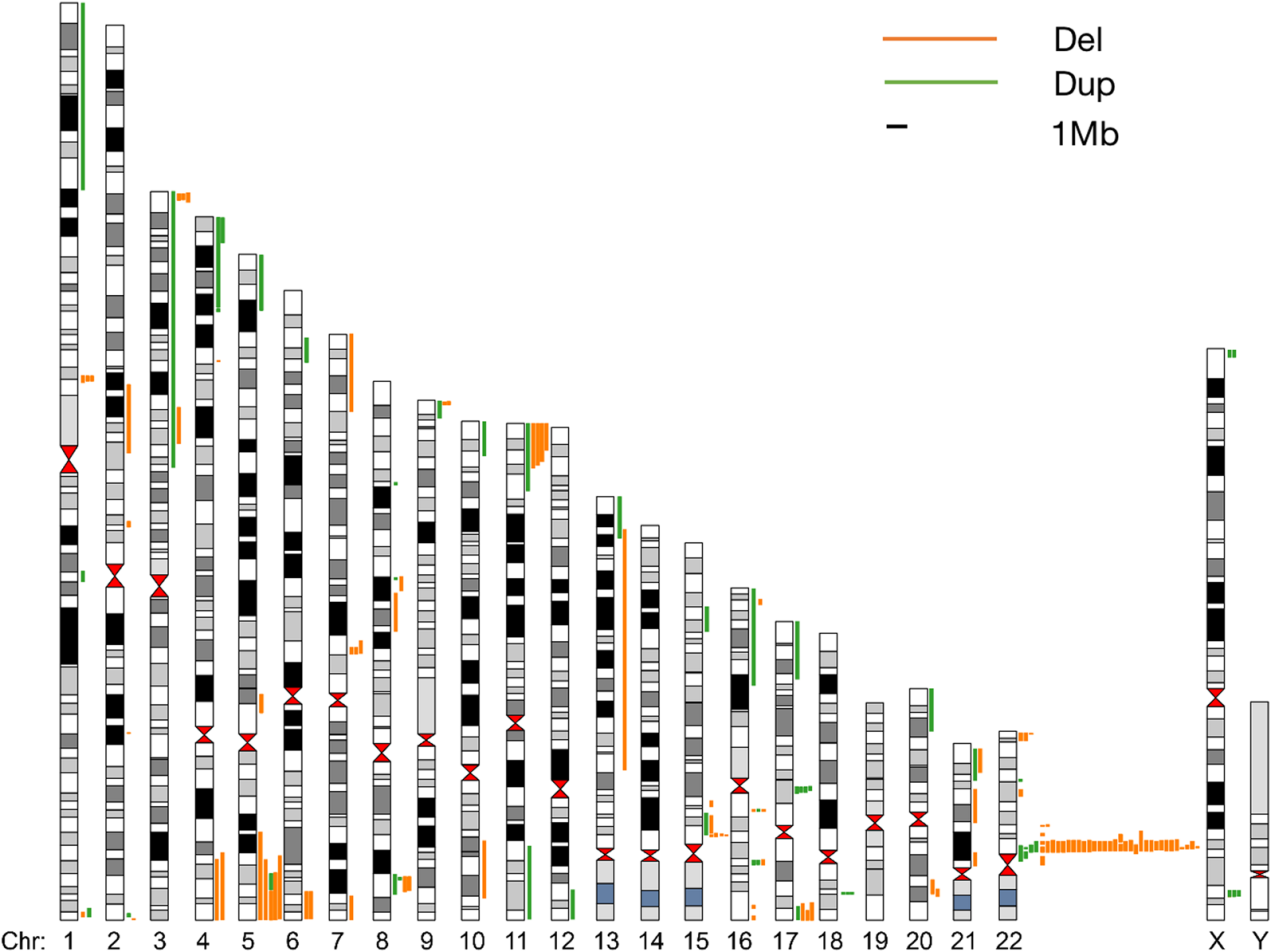
Hotspot significant CNVs related to CHD detected in 1585 Chinese by CMA. Dup: duplication; Del: deletion

We summarized and analysed CMA data from 200 cases in our study and 1,385 cases reported in 5 other literature reports (Table 6). A total of 161 P or LP CNVs were found in 9.0% of cases (143/1585). All chromosomes, except 14, 19, and Y, presented with clinically significant CNVs, and the clinically significant CNVs on chromosomes 22, 16, and 15 were the most common. Deletion of 22q11.2 was the most common clinically significant CNV, accounting for 27.3% (44/161). In foetuses with 22q11.2 deletion, the most common heart defects were Tetralogy of Fallot (52.3%, 23/44), ventricular septal defect (27.3%, 12/44), and interrupted aortic arch (18.2%, 8/44). The other 5 most commonly recurrent CNVs loci related to CHD were deletions of 5p15.33p15.31 (Cri du chat syndrome), deletions of 15q13.2q13.3 (Angelman/Prader–Willi syndrome), deletions of 11q24.2q25 (Jacobsen syndrome), deletions of 17p13.3p13.2 (Miller–Diekers syndrome), and duplications of 17q12. In addition, Fig. 2 showed all of the clinically significant CNVs from our study and the literatures. All CNVs found using the CMA in this cohort are listed in Table 7.

**Table 6:** The comparison of studies in prevalence of genetic variants identified in CHD fetuses by CMA

Study	Platform	Number	Total chromosomal abnormalities	Aneuploidies	Partial Aneuploidies	pCNVs	lpCNVs	Vous
Song et al. 2018 [18]	Affymetrix Cytoscan 750 k array	207	35 (16.9%)	17 (8.2%)	-	13 (6.3%)	5 (2.4%)	14 (6.8%)
Luo et al. 2018 [19]	Illumina HumanCytoSNP-12 v2.1 BeadChip	362	140 (38.7%)	111 (30.7%)	10 (2.8%)	17 (4.7%)	2 (0.6%)	-
Zhu et al. 2016 [20]	AffymetrixCytoScanplatform	115	21 (18.3%)	6 (5.2%)	2 (1.7%)	11 (9.6%)	2 (1.7%)	-
Wang et al. 2017 [21]	Illumina HumanCytoSNP-12 v2.1 BeadChip	602	133 (22.1%)	65 (10.8%)	20 (3.3%)	40 (6.7%)	8 (1.3%)	36 (6.0%)
Liao et al. 2014 [22]	Affymetrix CytoScan HD arrays	99	19 (19.2%)	excluded by CK	excluded by CK	19 (19.2%)	-	13 (13.1%)
Our study	Affymetrix Cytoscan 750 k array	200	49 (24.5%)	23 (11.5%)	-	20 (10.0%)	6 (3.0%)	8 (4%)

CK: conventional karyotype; pCNVs: pathogenic copy number variation; lpCNVs: likely pathogenic copy number variation

**Table 7:** Pathogenic or likely pathogenic CNVs found in the cohort

CASE	CHD Phenotype	Soft markers	Structural anomalies	CNV	Microarray Nomenclature	Size (Mbp)	Pathogenicity	Some of Relevant Genes	syndrome
#6	VSD,	–	Facial dysmorphisms	del	arr[hg19] 3q29(194,654,896–197,363,564) × 1	2.71	P	*TFRC*	Chromosome 3q29 microdeletion syndrome
#20	TOF	–	Respiratory system	del	arr[hg19] 22q11.1q11.21(17,900,000–20,600,000) × 1	2.7	P	*ATP6V1E1, PEX26,TUBA8*	22q11 deletion syndrome
#23	PVS	–	–	del	arr[hg19] 2p25.3(50–450,000) × 1	0.45	P	–	–
#24	Complex CHD	–	–	dup	arr[hg19] 15q24.3q25.2(78,250,000–85,000,000) × 1	6.75	P	*ARNT2, RPS17,AP3B2*	Chromosome 15q25 deletion syndrome
#35	Complex CHD	–	–	del	arr[hg19] 5p15.33p14.2(20,000–23,980,000) × 3	23.96	P	*SDHA, NDUFS6*	Mitochondrial complex II deficiency, Leigh syndrome(LS)
#35	Complex CHD	–	–	dup	arr[hg19] 21(q22.12-q22.3)(37,780,000–48,100,000) × 3	10.32	P	–	Down syndome
#39	PAA	–	–	del	arr[hg19] 7q11.23(72,260,000–76,000,000) × 3	3.74	P	*POR*	7q11.23 duplication syndrome, WILLIAMS-BEUREN region duplication syndrome
#41	d-TGA	–	–	dup	arr[hg19] 1p36.33p36.32(820,000–3,320,000) × 3	2.5	LP	*AGRN,B3GALT6, TMEM240*	congenital myasthenic syndrome-8 (CMS8), Ehlers-Danlos syndrome progeroid type 2
#56	DORV, PAA	–	–	del	arr[hg19] 5p15.33p15.1(20,000–16,500,000) × 1	16.48	P	*SDHA*	5p15 terminal (Cri du chat syndrome) region duplication,mitochondrial complex II deficiency[OMIM:252011]
#56	DORV,PAA	–	–	dup	arr[hg19] 17q24.2q25.3(65,320,000–81,160,000)) × 3	15.84	P	*PSMD12*	46 XX sex reversal 2, STANKIEWICZ-ISIDOR syndrome(STISS)
#59	AVSD	–	–	del	arr[hg19] 20p12.3(6,300,001–8,580,000) × 3	2.28	LP	*BMP2*	BRACHYDACTYLY, TYPE A2(BDA2), Early infantile epileptic encephalopathy-12 (EIEE12)
#78	VSD + ASD	Single umbilical artery	Facial Dysmorphisms	del	arr[hg19] 7p22.3p22.1(40,000–6,740,000) × 1	6.7	LP	BRAT1	lethal neonatal rigidity and multifocal seizure syndrome
#78	VSD + ASD	Single umbilical artery	Facial dysmorphisms	dup	arr[hg19] 12p13.33p13.31(160,000–8,320,000) × 3	8.16	LP	CACNA1C, CCND2, CHD4	Timothy syndrome(TS), SIFRIM-HITZ-WEISS syndrome (SIHIWES)
#82	TOF	–	–	dup	arr[hg19] 8p23.1(6,920,000–12,580,000) × 3	5.66	P	–	8p23.1 duplication syndrome
#84	TOF	–	–	del	arr[hg19] 22q11.21(18,880,000–21,440,000) × 1	2.56	P	*PRODH*	DIGEORGE syndrome; DGS (proximal, A-B)syndrome1,
#85	VSD, IAA	–	–	del	arr[hg19] 22q11.21q11.23(18,564,800–24,300,000) × 1	3.2	P	*PEX26, TUBA8,PRODH*	22q11 deletion syndrome
#100	TOF	–	–	del	arr[hg19] 22q11.21(18,970,561–21,800,471) × 1	2.8	P	*CLTCL1, HIRA,TBX1*	DiGeorgesyndrome(DGS), velocardio facial syndrome(VCFS)
#110	Other CHD	–	Central nervous	del	arr[hg19] 7q33q36.3(137,754,586–159,119,707) × 1	21.4	P	*BRAF, CNTNAP2, DPP6*	Cardiofaciocutaneous syndrome-1, Noonan syndrome 7,
#110	Other CHD	–	Central nervous	dup	arr[hg19] 20q13.2q13.33(51,222,942–62,913,645) × 3	11.7	P	*CYP24A1, PCK1,OSBPL2, RTEL1*	HYPERCALCEMIA INFANTILE-1(HCINF1), phosphoenolpyruvate carboxykinase deficiency
#111	VSD	–	–	del	arr[hg19] 16p11.2(29,428,531–30,190,029) × 1	0.74	LP	*PRRT2, KCTD13, TBX6, MAPK3*	16p11.2deletion syndrome
#114	AS	–	Skeletal system	del	arr[hg19] 22q11.21(18,631,364–21,800,471) × 1	3.2	P	*CLTCL1,HIRA,TBX1*	DiGeorgesyndrome(DGS), velo- cardio- facial syndrome(VCFS)
#116	CA, VSD, PLSVC	–	Cystic hygroma, Skeletal system	del	arr[hg19] 4p16.3p15.31(68,345–18,451,423) × 1	18.4	P	*LONP1, NFIX, SMARCA4*	BWS/SRS (Beckwith–Wiedemann syndrome/Silver-Russell syndrome;CODAS syndrome
#116	CA, VSD, PLSVC	–	Cystic hygroma, Skeletal system	dup	arr[hg19] 11p15.5p15.1(230,680–20,167,667) × 3	19.9	P	*APC2,TICAM1, LONP1*	Pigmented nodular adrenocortical diseaseprimarysyndrome; CODAS syndrome
#117	Other CHD	Echogenic bowel	Skeletal system	del	arr[hg19] 15q11.2(22,770,421–23,277,436) × 1	0.50	LP	*TUBGCP5, CYFIP1, NIPA2, NIPA1*	15q11.2 recurrent region (BP1-BP2) (includes NIPA1) deletionsyndrome1
#120	Other CHD	Choroid plexus cysts	Facial dysmorphisms	dup	arr[hg19] Xp22.31(6,455,151–8,135,568) × 3	1.68	P	*PUDP, STS, VCX, PNPLA4*	Steroid sulphatase deficiency (STS)
#121	PAA,ASD	–	Urinary tract system, facial dysmorphisms	del	arr[hg19] 4q31.3(151,335,416–151,834,016) × 1	0.5	LP	*LRBA, MAB21L2*	–
#135	CA	–	–	del	arr[hg19] 21q22.2q22.3(39,985,071–48,093,361) × 1	8.1	P	*KPTN*	Autosomal recessive mental retardation-41 (MRT41)
#136	VSD	–	–	dup	arr[hg19] 9q34.11q34.3(133,343,703–141,018,648) × 3	7.7	P	–	–
#138	AVSD	–	–	del	arr[hg19] 16q24.1q24.2(85,520,919–87,149,214) × 1	1.6	P	*FOXF1, FOXC2, FOXL1*	–
#157	VSD, IAA	–	–	del	arr[hg19] 22q11.21(18,636,749–21,800,471) × 1	3.16	P	*CLTCL1, HIRA,TBX1*	DiGeorgesyndrome(DGS), velo- cardio- facial syndrome(VCFS)
#179	VSD	–	–	del	arr[hg19] 15q11.2(22,770,421–23,625,785) × 1	0.83	LP	*TUBGCP5, CYFIP1, NIPA2, NIPA1*	15q11.2 recurrent region (BP1-BP2)deletionsyndrome
#179	VSD	–	–	del	arr[hg19] 16p13.11(14,910,158–16,520,463) × 1	1.6	P	*NOMO1, NPIPA1, PDXDC1, NTAN1), RRN3, KIAA0430, NDE1, MYH11, ABCC1, ABCC6, NOMO3*	16p13.11 recurrent microdeletion syndrome

CNVs found by CMA in the cohort, with the number of genes present in the region, listing the most relevant genes and phenotypes for each individual. Dup, Duplication; Del, Deletion; CA, congenital anomalies; LP, likely pathogenic; P, pathogenic; VOUS, variants of uncertain significance. VSD, ventricular septal defect; TGA, transposition of the great arteries; CA, coarctation of aorta; RAA, right aortic arch; ASD, atrial septum defect; AS, aortic stenosis; AVSD, atrioventricular septal defect; TOF, tetralogy of fallot; HLHS, hypoplastic left heart syndrome; IAA, interruption of aortic arch; PLSVC: persistent left superior vena cava; PAA: pulmonary artery atresia; PVS: pulmonary valve stenosis; DOLV: double outlet right ventricle

## Discussion

In this study, we used a combination of the CMA and WES analysis to assess the prevalence of genetic diagnoses in foetuses with CHD. Chromosomal abnormalities were found using CMA in 24.5% of CHD foetuses. In addition, the additional diagnostic yield of clinical sequence variants by WES testing was 11.5% (6/52). Many studies have reported the detection rate of CMA in foetuses with CHD; however, the detection rate of CMA varies due to the different array types used [21]. It is reported that the positive rate of CMA test in prenatal evaluation is 6.6–38.7% [22, 23]. Overall, the yield of the CMA test in our study was consistent with that of previously published data. However, the detection rate of our pathogenic sequence variants was higher than that reported in existing literature [24–26]. We suggest CMA as a first-tier test in foetuses with CHD, and that WES can be offered sequentially if CMA results are negative.

It is widely believed that if there is an additional deformity in the foetus with CHD, it will significantly increase the possibility of potential genetic causes [27, 28]. In this study, the chromosomal abnormalities detection rates for isolated CHD, non-isolated CHD, CHD with structural anomalies, CHD with soft markers, and CHD with structural anomalies and soft markers were 20.9%, 31.8%, 28.6%, 27.3%, and 43.8%, respectively. The detection rate of non-isolated CHD foetuses was higher than that of isolated CHD foetuses (31.8% vs. 20.9%); however, the difference was not statistically significant (P > 0.05). This result was consistent with what was reported by Liao et al. [29]. However, we did observe that the incidence of chromosomal abnormalities was significantly high when CHD was combined with structural abnormalities and soft markers. Also, in our study, the detection rate in the complex CHD group (31.8%) was higher than, but not statistically different from that of the simple CHD group (23.6%). Rivka's study also showed that there was no significant difference in the incidence of chromosomal abnormalities between simple and complex CHD [30]. This suggests that the complexity of heart defects is not related to the frequency of chromosomal abnormalities. Therefore, we suggest that a CMA is the recommended initial examination for cases of CHD in the prenatal setting, including simple heart disease and isolated heart disease.

Furthermore, our results showed that compared with other types of structural abnormalities, the incidence of chromosomal abnormalities in CHD with central nervous system abnormalities was significantly increased; this phenomenon was also reported by Richards [31]. Previous studies have reported that soft markers indicate an increased risk of foetal chromosomal aneuploidy. In our study, the detection rate of aneuploidies in the CHD with soft markers group was as high as 22.7%, which is significantly higher than that in the isolated CHD group and the CHD with extracardiac structural anomalies group. Among all CHD groups, the highest detection rate of chromosomal abnormalities was found in those with AVSD (54.5%), followed by complex CHD (31.8%) and conotruncal defects (24.5%). Similar results have been obtained in previously published data. Wang et al. reported that among 602 cases of CHD detected using the CMA, the most common chromosomal abnormalities were observed in foetuses with AVSD (73.7%) [29]. Although the subgroup size of our cohort was small, we got very similar results as those of Wang's study.

We not only analysed the data from our centre for this cohort study but also systematically reviewed the literatures on clinically significant CNVs reported using the CMA in Chinese CHD foetuses. In our study, the overall detection rate of chromosomal abnormality was 24.5% (49/200), and the total detection rate for clinically significant CNVs was 13.0% (26/200). Moreover, the detection rate of total chromosomal abnormalities in 1,385 CHD foetuses summarized in the literatures was 25.1% (348/1385), and the detection rate of clinically significant CNVs was 8.4% (117/1385). Overall, the incidence of P chromosomal abnormality and clinically significant CNVs in CHD foetuses in our study were similar to what has been reported in the literatures. Studies have shown that the deletion of 22q11.2 is associated with cardiac abnormalities. Both in our study and in the literature, the most common CNV detected in CHD foetuses was in the 22q11.2 region. Additionally, 22q11.2 deletion was reported to be associated with Tetralogy of Fallot by Mercer-Rosa et al. [32]. In our study, 52.3% of foetuses with 22q11.2 deletions presented with Tetralogy of Fallot. In the combined data from the literature, 47.7% of foetuses with 22q11.2 deletions presented with Tetralogy of Fallot.

This study not only complements the previous data that CMA plays an important role in prenatal detection of CHD but also evaluated the yield of CMA in regard to specific clinical characteristics, and even discusses the clinical value of WES in prenatal diagnosis of CHD.

## Conclusions

CMA is the recommended initial examination for cases of CHD, including simple heart disease and isolated heart disease in prenatal settings. The follow-up application of WES will offer a considerable proportion of additional detection of clinical significance. 22q11.2 was the most common pathogenic region of the Chinese population.

## Methods

### Participants

This cohort study aimed to assess the prevalence of chromosomal abnormalities in Chinese foetuses with different types of CHD and was carried out in the department of medical genetics of Changzhou Maternal and Child Health Hospital between 1st January 2015 and 31st August 2020. The main inclusion criteria were: (1) Foetuses who were diagnosed with CHD with or without other structural anomalies or soft markers using prenatal ultrasound; (2) The pregnant women who voluntarily chose to undergo a CMA to determine the etiological diagnosis; (3) Both parents agreed to conduct a further WES analysis and provided their samples; and (4) The quantity and quality of foetal DNA were qualified. Foetuses with the following ultrasound findings were excluded from the study: isolated persistent left superior vena cava or valve insufficiency, coronary artery malformation, or heart tumour. Pregnant women whose foetuses presented with heart defects were referred to our prenatal diagnostic centre for further genetic testing and counselling. Couples who volunteered for prenatal CMA diagnosis signed written informed consent and underwent invasive prenatal diagnostic procedures. Cases with a positive CMA result received genetic counselling again. WES was subsequently performed in some cases with negative CMA test results. All foetal specimens were amniotic fluid cells. The average age of the pregnant women was 28.26 years (17–46 years), and the average gestational age of foetus was 25.5 weeks (22–30 weeks). Most pregnant women did not receive serological prenatal screening and/or noninvasive prenatal screening. They directly received prenatal diagnosis after amniocentesis because of foetal ultrasound abnormalities in the second trimester.

Of the 200 foetuses with CHD, 134 with only abnormalities of the heart were classified as the isolated CHD group, and 66 with other extracardiac ultrasound abnormalities, including structural abnormalities and soft markers, were classified as the non-isolated CHD group. Soft markers in this study included increased nuchal folds (≥ 6.0 mm), nuchal translucency (≥ 3.0 mm), echogenic bowels, mild ventriculomegaly (10–15 mm), persistent right umbilical veins, absent nasal bones, and a single umbilical artery. The detailed anatomical classification of CHD in this study was based on the method described by Botto et al. [14]. Moreover, cardiac defects were defined as simple CHD (n = 178) if they were anatomically discrete or single or dominant entities and complex CHD (n = 22) if they were characterized by transposition of great arteries, single ventricle or multiple cardiac abnormalities (involving three or more defects).

### CMA

The CMA was performed using affymetrix Cytoscan 750 k array platform. Genomic DNAs were extracted from collected amniotic fluid using the QIAamp DNA Micro Kit. After polymerase chain reaction amplification, DNA was digested and ligated to adapters, and subsequently array hybridization, washing, and scanning were performed. Data was analysed using the Affymetrix Chromosome Analysis Suite software, based on genome version GRCh37 (hg19). We used the following public databases: PubMed (http://www.ncbi.nlm.nih.gov/pubmed/), ISCA (https://www.iscaconsortium.org/), UCSC (http://genome.ucsc.edu), OMIM (http://www.ncbi.nlm.nih.gov/omim), DECIPHER (http://decipher.sanger.ac.uk/), and DGV (http://www.ncbi.nlm.nih.gov/dbvar/). According to the American College of Medical Genetics guidelines [15], all CNVs reported in this study were classified as VOUS, LP, or P. Both LP and P CNVs were considered as clinically significant CNVs in this study.

### WES analysis

Genomic DNA samples were extracted from amniocytes using a Qiagen DNA Blood Midi/Mini kit (Qiagen, Valencia, CA, USA) according to instruction. Agilent SureSelect Human All Exon capture kit (Exome V6) was used for exomes capture and HiSeq2500 sequencer was used for two paired-end massively parallel sequencing.

Similar to previously used methods [16–18], the bcl2fastq software (Illumina) was used to process Raw image files, make base calling, and generate raw data. We used Trimmomatic to filter adapter contaminated and low-quality reads (quality threshold 20). Next, we aligned the filtered clean sequencing reads to the National Centre for Biotechnology Information human reference genome (hg19) through Burrows–Wheeler Aligner. Thereafter, a single nucleotide polymorphism analysis, repeated labelling, indel rearrangement, and the recalibration of BAM files were performed using SAM tools and GATK.

After using Exome Variant Server databases (http://evs.gs.washington.edu/EVS), dbSNP (http://www.ncbi.nlm.nih.gov/snp), 1000 Genomes Project (1000 GP) (http://browser.1000genomes.org), and Genome Aggregation Databases (gnomAD) (http://gnomad.broadinstitue.org/) minor allele frequencies (MAFs) of all known variants were reported. The perniciousness and pathogenicity of mutations were determined by the following databases: OMIM (http://www.omim.org), ClinVar (http://www.ncbi.nlm.nih.gov/clinvar), and Human Gene Mutation Database (Professional 2019) (http://www.hgmd.org). In order to analyse the biological effects and evaluate the pathogenicity of all the detected variants, the following prediction softwares were used: SIFT (http://sift.jcvi.org), Mutation Taster (http://www.mutationtaster.org), PolyPhen-2 (http://genetics.bwh.harvard.edu/pph2), PROVEAN (http://provean.jcvi.org/index.php vean.jcvi.org/index.php), CADD (http://cadd.gs.washington.edu), Human Splicing Finder (http://www.umd.be/HSF), and MaxEntScan (http://genes.mit.edu/burgelab/maxent/Xmaxentscan_scoreseq.html).

As for quality control standards, we required an average sequencing depth of ≥ 100 × and a sequencing coverage of 20 ×  ≥ 99% for each sample. First, in the analysis, variants were selected according to the following criteria: (1) sequencing depth ≥ 20, alternative allele frequency ≥ 0.3; (2) in dbSNP build 150, 1000 GP, and gnomAD, the MAF < 0.01; (3) variants located in coding regions or exon–intron junctions; (4) predicting harmful proteins or splicing by in silico algorithms; and (5) gene variants associated with OMIM disease/phenotype. Subsequently, the variants were further selected according to the disease inheritance models. Finally, according to the guidelines of the American College of Medical Genetics and Genomics [19], all the above selected variants were classified as P, LP, VOUS, likely benign, or benign. In addition, WES data were analysed in all cases according to the clinical indications of invasive prenatal diagnosis. Sanger sequencing was used to confirm all the reported variants, and the specific primer sequences are shown in Additional file 1: Table S1.

### Systematic literature search

To investigate hotspot pathogenic CNVs associated with CHD in the Chinese population, we performed a systematic literature search according to the Preferred Reporting Items for Systematic Reviews and Meta-Analyses guidelines [20], when applicable. We searched PubMed for all published cohort studies that reported CMA results of foetal CHD from February 2015 to February 2020 using the following search string: [(“CHD” OR “congenital heart defect” OR “congenital heart disease”) AND (“CMA” OR “CNVs” OR “genetic diagnoses” OR “chromosomal microarray analysis” OR “copy number variants” OR “Genetic Testing”)] NOT review [pt] AND English [la].

Studies were included if they met the following criteria: (1) More than 99 samples were included in the study, (2) the participants of the study were Chinese and (3) detailed chromosomal loci of P and LP CNVs were clearly described.

### Statistical analyses

SPSS software (IBMSPSS statistics version 14.0) was used for the statistical analysis. The chromosomal abnormalities or aneuploidies or CNVs detection rates in isolated CHD, non-isolated CHD, simple CHD, and complex CHD were pairwise compared using fisher’s exact test or the chi square test. In all tests, P < 0.05 was defined as statistically significant.

## Supplementary Information

**Additional file 1.** **Supplementary table 1.** The specific primer sequences of Sanger sequencing.

## Data Availability

The questionnaire and datasets used are available from the corresponding author on request.

## Abbreviations

AVSD: Atrioventricular septal defects
CHD: Congenital heart defects
CMA: Chromosome microarray analysis
CNVs: Copy number variations
LP: Likely pathogenic
MAF: Minor allele frequencies
P: Pathogenic
VOUS: Variant of unknown significance
WES: Whole exome sequencing
