# Supplementary material for: Estimating the frequency of causal genetic variants in foetuses with congenital heart defects: a Chinese cohort study
Source: Orphanet J Rare Dis. 2022 Jan 4;17:2. doi: 10.1186/s13023-021-02167-8 (PMC8729135; doi:10.1186/s13023-021-02167-8)
Supplement: Supplementary file 1 — Additional file 1. Supplementary table 1. The specific primer sequences of Sanger sequencing. [file 13023_2021_2167_MOESM1_ESM.docx]

Supplementary table 1. The specific primer sequences of Sanger sequencing

| **Gene** | **Nucleotide change** | **Chromosome location** | **Primer sequences** |
| --- | --- | --- | --- |
| *SCN5A* | c.4357C>T | chr3:38597170 | F:5-AGGCTGGGCTGAAAGACT R:5-TCAATCCTGGCATCCTCA |
| *NIPBL* | c.5220delA | chr5:37020770 | F:5-TGGTTTCGAGACACAACT R:5-ATCAGAGTTCATCCTGCA |
| *FOXF1* | c.849_850delAT | chr16:86545024 | F:5-CGTCCAAGGCCAAGAAGAC R:5-CAAACTAGGGTCAAGAGCGG |
| *SCN5A* | c.362G>A | chr3:38671832 | F:5-CTCTGAGCCAAAAGCTGCC R:5-CACGTGCACATCCCCAG |
| *GATA6* | c.551G>A | chr18:19751656 | F:5-GTCGAGCTGGGAGGACTTG R:5-GGGAGAGTAGGGGAAGCG |
| *MYH7* | c.4076G>A | chr14:23892874 | F:5-TCACATCATCCACTTGCT R:5-ACCATACTGACCTTGACC |
| *CITED2* | c.589A>G | chr6:139694508 | F:5-CCAAACCCATTTCTATCACC R:5-CAACCACTACATGCCGGATT |
| *NOTCH1* | c.7171C>T | chr9:139391020 | F:5-TGGCTCGGCTCTCCACTC R:5-GTGCCGAACCAATACAACCC |
| *NOTCH1* | c.5339_5346dupAGAAGAAG | chr9:139396761 | F:5-GGAGGAGAGTGGGTGAGGAG R:5-GTCCAGGTCAGGCAGAACC |
| *COL9A3* | c.622G>A | chr20:61456365 | F:5-ACTTCAGTGTTGCCAGGGAG R:5-GAGCCCAGCCTCCAGTC |
| *PRNP* | c.622C>T | chr20:4680488 | F:5-AGTCAGTGGAACAAGCCGAG R:5-AAGGGCTGCAGGTGGATAC |
| *HDAC* | c.1+1G>A | chr2:240112674 | F:5-CTGTTTTCTCAGCAGCCCTC  R:5-TCTGCCCTGTACTAATGTGCT |
| *DNAH9* | c.1997G>T | chr17:11550415 | F:5-CAGATCATGACCAGGCACC R:5-GAAATCAGCAGAGTTCAAAGAAAC |
| *DNAH9* | c.14_34delAGGAGCGGGCCGCGC | chr17:11501820 | F:5-CAGAGGAGTGAGTCCTGGC R:5-CTTAAGGCTCCTCCTCCCC |
